# Supplementary material for: Dairy Consumption and 3-Year Risk of Type 2 Diabetes after Myocardial Infarction: A Prospective Analysis in the Alpha Omega Cohort
Source: Nutrients. 2021 Sep 9;13(9):3146. doi: 10.3390/nu13093146 (PMC8467112; doi:10.3390/nu13093146)
Supplement: Supplementary file 1 [file nutrients-13-03146-s001.zip › nutrients-1342916-supplementary.pdf]

**Supplementary Files**

**Article:** "Dairy Consumption and 3-year Risk of Type 2 Diabetes after Myocardial Infarction: A Prospective Analysis in the Alpha Omega Cohort"

**Authors:** Jacobo-Cejudo, M.G.; Cruijsen, E.; Heuser, C.; Soedamah-Muthu, S.S.; Voortman, T. & Geleijnse, J.M.

**Contact:** maria.jacobocejudo@wur.nl or ln_gorejace@yahoo.com.mx

**Supplemental Figure S1** **(page 3)**
Participant selection for the analyses of dairy consumption and 3-y risk of T2D in 3401 patients of the Alpha Omega Cohort in the Netherlands.

**Supplemental Figure S2** **(page 4)**
Participant selection for the analyses of dairy consumption and 3-y (40 months) changes in body weight, body mass index (BMI) and waist circumference in 2313 patients of the Alpha Omega Cohort in the Netherlands.

**Supplemental Table S1** **(page 5)**
Classification of dairy foods in the Alpha Omega Cohort.

**Supplemental Table S2** **(page 6)**
Baseline dietary intake of 3401 post-MI patients from the Alpha Omega Cohort, overall and across categories of energy-adjusted total milk consumption.

**Supplemental Table S3** **(page 7)**
HRs (95% CIs) for type 2 diabetes with full-fat milk, full-fat yogurt, liquid fermented dairy, and (ice)-cream intake in the Alpha Omega Cohort

**Supplemental Table S4** **(page 8)**
HRs (95% CIs) for type 2 diabetes according to categories of types of dairy consumption in the Alpha Omega Cohort, without adjustment for BMI.

**Supplemental Table S5** **(page 10)**
HRs (95% CIs) for type 2 diabetes with full-fat milk, full-fat yogurt, liquid fermented dairy, and (ice)-cream intake in the Alpha Omega Cohort, without adjustment for BMI.

**Supplemental Table S6** **(page 11)**
Longitudinal changes in anthropometric outcomes after 3-y of follow-up in 2313 patients from the Alpha Omega Cohort.

**Supplemental Table S7** **(page 12)**
Beta coefficients (95% CIs) for 3-y changes in anthropometric outcomes according to categories of types of dairy consumption in 2313 patients from the Alpha Omega Cohort.

**Supplemental Table S8** **(page 14)**
HRs (95% CIs) for type 2 diabetes according to categories of types of dairy consumption among patients with obesity (n = 685).

**Supplemental Table S9** **(page 16)**
HRs (95% CI) for type 2 diabetes according to categories of types of dairy consumption among patients without obesity (n = 2716).

**Supplemental Table S10** **(page 18)**
HRs (95% CIs) for type 2 diabetes according to categories of types of dairy consumption among women (n=680).

**Supplemental Table S11** **(page 20)**
HRs (95% CIs) for type 2 diabetes according to categories of types of dairy consumption among men (n=2721).

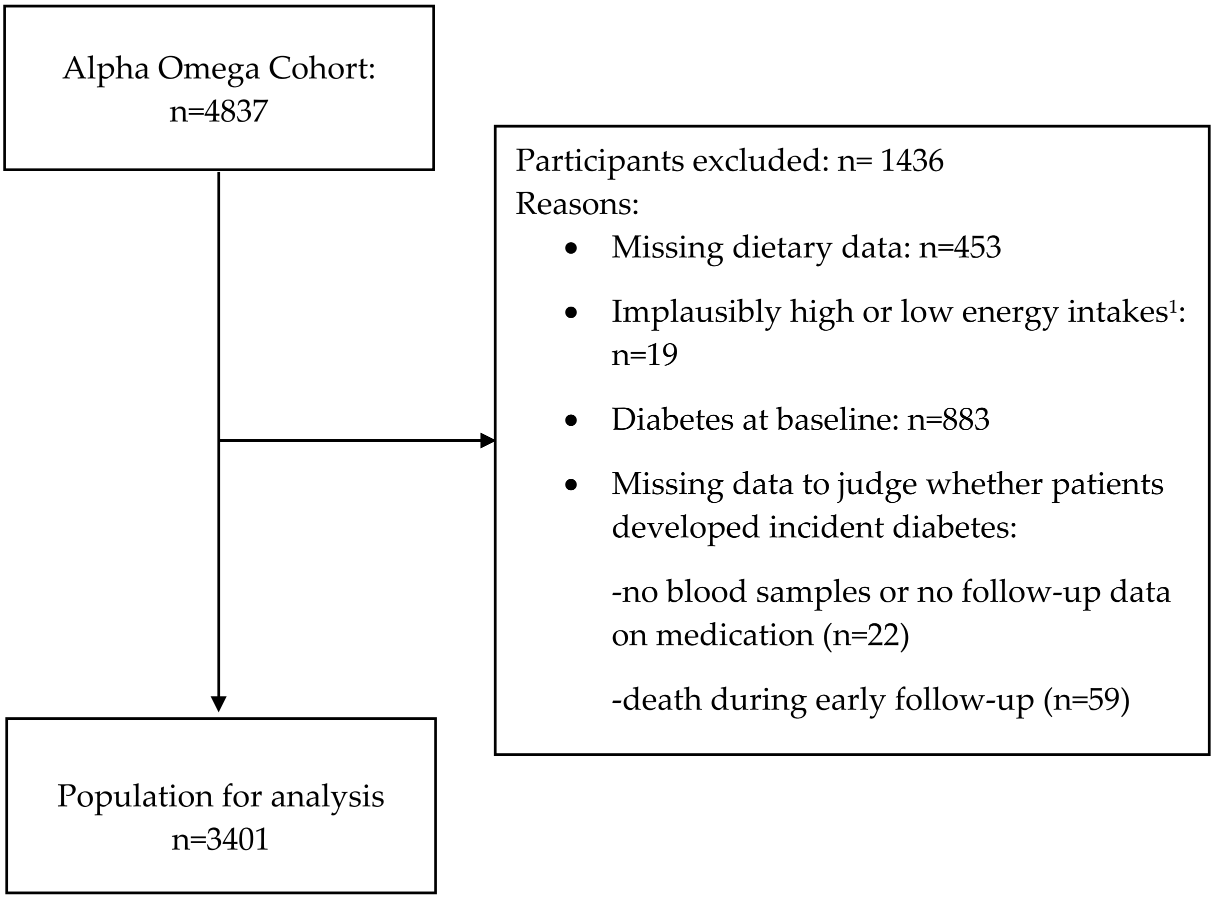


**Supplemental Figure S1**. Participant selection for the analyses of dairy consumption and 3-y risk of T2D in 3401 patients of the Alpha Omega Cohort in the Netherlands. [1]Implausibly high or low energy intakes: for women <600 and >6000 kilocalories per day and for men <800 and >8000 kilocalories per day.

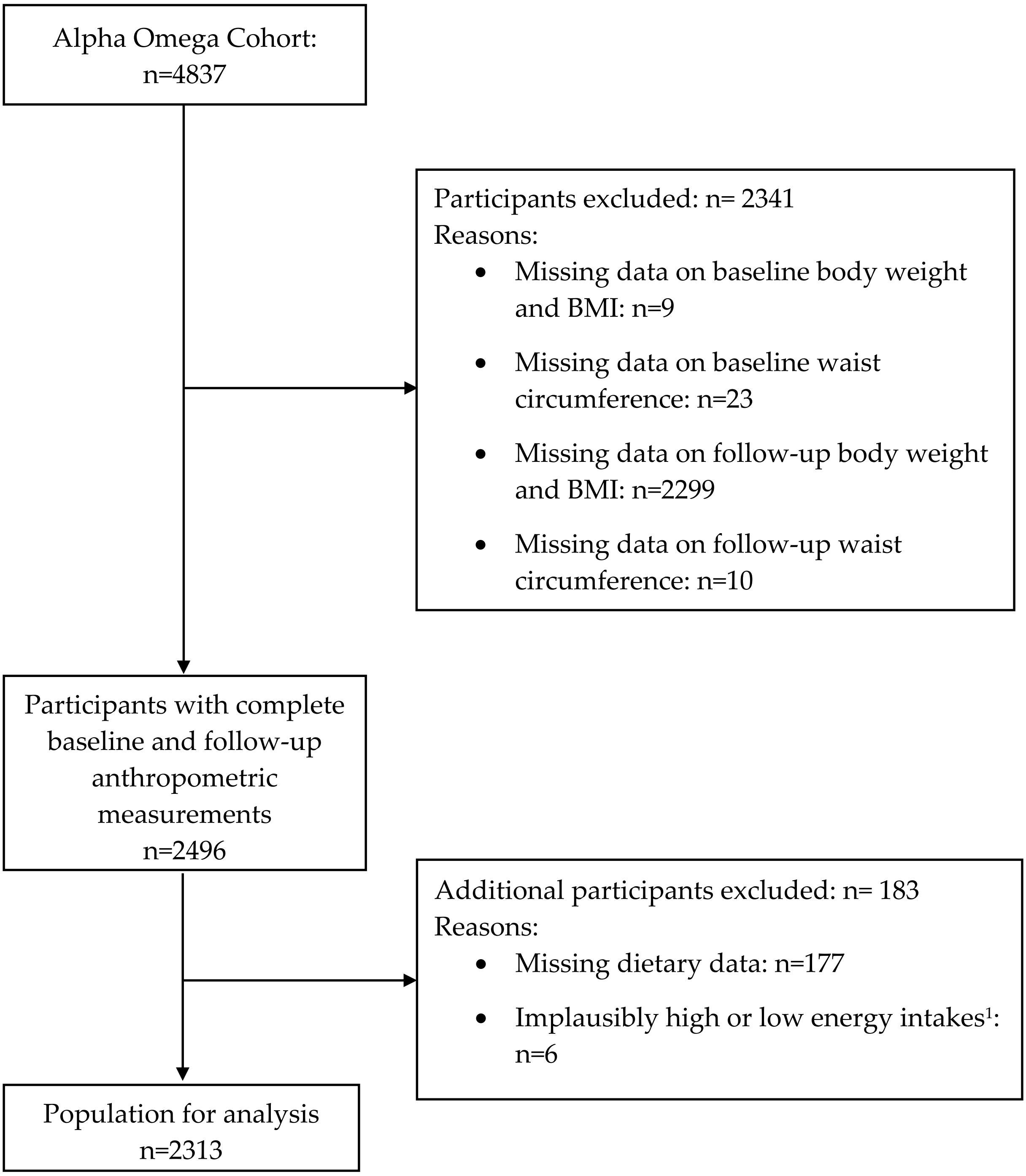


**Supplemental Figure S2.** Participant selection for the analyses of dairy consumption and 3-y (40 months) changes in body weight, body mass index (BMI) and waist circumference in 2313 patients of the Alpha Omega Cohort in the Netherlands. [1]Implausibly high or low energy intakes: for women <600 and >6000 kilocalories per day and for men <800 and >8000 kilocalories per day.

**Supplemental Table S1.** Classification of dairy foods in the Alpha Omega Cohort.

| Dairy food types | Dairy foods included |
|---|---|
| Total Dairy | Included total milk, soft cheese [full-fat luxury cheese (full-fat brie, soft cream cheese, blue cheese, Kernhem), reduced-fat luxury cheese (reduced fat brie, Camembert, Rambol, soft goat cheese), quark (cottage cheese), mozzarella, and cream cheese)], hard cheese, total yogurt, liquid fermented dairy, (ice)-cream, and dairy based desserts. |
| Total milk | All types of milk including low-fat milk, full-fat milk, and milk in coffee. |
| Low-fat milk | ≤2% fat milk (skimmed and semi-skimmed milk). |
| Full-fat milk | ≥3.5 % fat milk. |
| Hard cheese | Dutch regular 20% (spread cheese 20%, cottage cheese, Swiss spread cheese), 30% (spread cheese 30%, Cantenaar, Milner, Zaanlander light), 40% and 48% fat cheese (Gouda cheese from young to old, smoked cheese, Edam, Maasdam, Maaslander, Leerdammer, Padano, boerenkaas (farmer´s cheese), Emmental, Cheddar, hard goat cheese), parmesan, and gruyere. |
| Total yogurt | All types of plain yogurt including low-fat yogurt and full-fat plain yogurt. |
| Low-fat yogurt | Skimmed and semi-skimmed plain yogurt |
| Full-fat yogurt | Full-fat plain yogurt |
| Liquid fermented dairy | Buttermilk and yogurt drink |
| Total fermented dairy | Buttermilk, yogurt drink, yogurt, curd, hard and soft cheese. |
| (Ice)-cream | Ice cream, whipped cream and any type of cream. |
| Dairy based desserts | Flavored yogurt drink, chocolate milk, custard (vla), pudding and porridge. |

**Supplemental Table S2.** Baseline dietary intake of 3401 post-MI patients from the Alpha Omega Cohort, overall and across categories of energy-adjusted total milk consumption.[1]

| | Total population (n = 3401) | Categories of milk consumption (g/d) | | |
|---|---|---|---|---|
| | | <50 (n = 1303) | ≥50 – 150 (n =1075) | ≥150 (n=1023) |
| *Dairy intakes, g/d* | | | | |
| Total dairy[2] | 264 (173-391) | 187 | 255 | 442 |
| Total milk[2] | 82 (0-150) | 0 | 107 | 267 |
| Low-fat milk[2] | 64 (0-150) | 0 | 100 | 220 |
| Full-fat milk,%[2] | 10 (328) | 7 | 11 | 11 |
| Hard cheese[2] | 16 (8-28) | 17 | 16 | 15 |
| Total yogurt[2] | 41 (12-89) | 41 | 37 | 41 |
| Low-fat yogurt[2] | 32 (0-88) | 32 | 34 | 32 |
| Full-fat yogurt,%[2] | 30 (1003) | 33 | 27 | 27 |
| Liquid fermented,%[2] | 38 (1271) | 44 | 40 | 29 |
| Total fermented[2] | 154 (64-202) | 160 | 131 | 156 |
| (Ice)-cream,%[2] | 66 (2203) | 67 | 68 | 62 |
| Dairy based desserts[2] | 43 (21-78) | 45 | 41 | 42 |
| *Other dietary intakes, g/d* | | | | |
| Whole grains | 118 (88-158) | 124 | 120 | 105 |
| Refined grains | 41 (21-71) | 43 | 41 | 37 |
| Total potatoes | 99 (50-99) | 99 | 99 | 99 |
| Fruit | 110 (43-249) | 110 | 106 | 112 |
| Vegetables | 78 (58-103) | 78 | 80 | 76 |
| Total red and processed meat | 69 (43-94) | 72 | 66 | 67 |
| Sugar-sweetened beverages | 30 (0-108) | 28 | 38 | 28 |
| Total coffee | 375 (375-563) | 375 | 375 | 375 |
| Total tea | 150 (54-450) | 188 | 150 | 150 |
| Total energy intake, kcal/d | 1946 ± 528 | 2006 ± 511 | 1906 ± 550 | 1913 ± 518 |

[1]Values are mean ± SD for normally distributed variables, medians (IQRs) for skewed variables or %(n) of consumers for dairy types with relatively low intake (full-fat milk, full-fat yogurt, liquid fermented dairy and ice-cream). Data shown are original intakes across categories of energy-adjusted total milk intake. [2]Classification of dairy food types can be seen in supplemental table 1.

**Supplemental Table S3.** HRs (95% CIs) for type 2 diabetes with full-fat milk, full-fat yogurt, liquid fermented dairy, and (ice)-cream consumption in the Alpha Omega Cohort[1].

| | Dairy consumption | | |
|---|---|---|---|
| | No intake | Any intake | P-value |
| Full-fat milk | | | |
| Median intake, g/d | 0 | 53 | |
| Patients / events (n) | 3068/171 | 333/15 | |
| Person-years | 9673 | 1041 | |
| Model 1 | 1 | 0.82 (0.48-1.39) | 0.46 |
| Model 2 | 1 | 0.80 (0.47-1.36) | 0.41 |
| Model 3 | 1 | 0.79 (0.46-1.36) | 0.40 |
| Model 4 | 1 | 0.80 (0.47-1.38) | 0.43 |
| | | | |
| Full-fat yogurt | 0 | 15 | |
| Median intake, g/d | 2398/137 | 1003/49 | |
| Patients / events (n) | 7573 | 3142 | |
| Person-years | 1 | 0.86 (0.62-1.20) | 0.38 |
| Model 1 | 1 | 0.87 (0.63-1.22) | 0.44 |
| Model 2 | 1 | 0.87 (0.62-1.21) | 0.42 |
| Model 3 | 1 | 0.88 (0.63-1.23) | 0.46 |
| Model 4 | | | |
| | | | |
| Liquid fermented dairy | 0 | 75 | |
| Median intake, g/d | 2108/113 | 1293/73 | |
| Patients / events (n) | 6626 | 4088 | |
| Person-years | 1 | 1.04 (0.78-1.40) | |
| Model 1 | 1 | 1.01 (0.75-1.36) | 0.75 |
| Model 2 | 1 | 1.03 (0.76-1.38) | 0.92 |
| Model 3 | 1 | 1.03 (0.76-1.40) | 0.84 |
| Model 4 | | | 0.81 |
| | | | |
| (Ice)-cream | 0 | 13 | |
| Median intake, g/d | 1163/70 | 2238/116 | |
| Patients / events (n) | 3667 | 7047 | |
| Person-years | 1 | 0.85 (0.63-1.16) | |
| Model 1 | 1 | 0.84 (0.62-1.15) | 0.32 |
| Model 2 | 1 | 0.86 (0.63-1.17) | 0.27 |
| Model 3 | 1 | 0.86 (0.63-1.18) | 0.35 |
| Model 4 | | | 0.37 |

[1]Hazard ratios and 95% confidence intervals from Cox regression. Full-fat milk, full-fat yogurt, liquid fermented dairy and (ice)-cream) are modelled as any intake versus no intake. Model 1 was adjusted for age (y), sex and total energy intake (kcal/d). Model 2 was additionally adjusted for smoking (3 categories), physical activity (3 categories), educational level (3 categories), BMI (kg/m$^2$) and alcohol intake (4 categories). Model 3 was additionally adjusted for dietary intakes (g/d) of whole grains, refined grains, potatoes, fruit, vegetables, total red and processed meat, sugar-sweetened beverages, coffee, and tea. Model 4 was additionally adjusted for other dairy intakes.

**Supplemental Table S4.** HRs (95% CIs) for type 2 diabetes according to categories of types of dairy consumption without adjustment for BMI in the Alpha Omega Cohort[1].

| | Categories of dairy consumption (g/d) | | | |
|---|---|---|---|---|
| | Low | Intermediate | High | *P* for trend[2] |
| Total dairy | <200 | ≥200 - 400 | ≥400 | |
| Median intake, g/d | 137 | 283 | 527 | |
| Patients / events (n) | 1004/48 | 1559/86 | 838/52 | |
| Person-years | 3159 | 4923 | 2632 | |
| Model 2 | 1 | 1.17 (0.81-1.69) | 1.32 (0.88-1.97) | 0.19 |
| Model 3 | 1 | 1.22 (0.85-1.76) | 1.40 (0.93-2.10) | 0.11 |
| Total milk | <50 | ≥50 - 150 | ≥150 | |
| Median intake, g/d | 11 | 102 | 244 | |
| Patients / events (n) | 1303/65 | 1075/65 | 1023/56 | |
| Person-years | 4108 | 3366 | 3241 | |
| Model 2 | 1 | 1.21 (0.86-1.71) | 1.07 (0.75-1.54) | 0.74 |
| Model 3 | 1 | 1.24 (0.88-1.76) | 1.10 (0.76-1.58) | 0.64 |
| Model 4 | 1 | 1.25 (0.88-1.76) | 1.11 (0.77-1.60) | 0.61 |
| Low-fat milk | <50 | ≥50 - 150 | ≥150 | |
| Median intake, g/d | 10 | 104 | 249 | |
| Patients / events (n) | 1492/78 | 969/55 | 940/53 | |
| Person-years | 4693 | 3045 | 2976 | |
| Model 2 | 1 | 1.08 (0.76-1.53) | 1.06 (0.75-1.51) | 0.64 |
| Model 3 | 1 | 1.11 (0.78-1.57) | 1.09 (0.76-1.55) | 0.51 |
| Model 4 | 1 | 1.10 (0.78-1.57) | 1.09 (0.77-1.56) | 0.50 |
| Hard cheese | <15 | ≥15-30 | ≥30 | |
| Median intake, g/d | 9 | 21 | 48 | |
| Patients / events (n) | 1397/73 | 1174/71 | 830/42 | |
| Person-years | 4396 | 3694 | 2624 | |
| Model 2 | 1 | 1.16 (0.83-1.63) | 0.96 (0.66-1.41) | 0.80 |
| Model 3 | 1 | 1.17 (0.83-1.64) | 0.98 (0.67-1.44) | 0.87 |
| Model 4 | 1 | 1.16 (0.83-1.64) | 0.98 (0.66-1.44) | 0.87 |
| Total yogurt | <25 | ≥25-50 | ≥50 | |
| Median intake, g/d | 8 | 36 | 86 | |
| Patients / events (n) | 1271/70 | 635/31 | 1495/85 | |
| Person-years | 4008 | 1999 | 4707 | |
| Model 2 | 1 | 0.89 (0.58-1.37) | 1.05 (0.76-1.46) | 0.64 |
| Model 3 | 1 | 0.91 (0.59-1.40) | 1.09 (0.78-1.52) | 0.51 |
| Model 4 | 1 | 0.91 (0.59-1.41) | 1.07 (0.77-1.49) | 0.59 |

| | | | | |
|---|---|---|---|---|
| Low-fat yogurt | <25 | ≥25-50 | ≥50 | |
| Median intake, g/d | 5 | 37 | 90 | |
| Patients / events (n) | 1555/81 | 473/22 | 1373/83 | |
| Person-years | 4895 | 1509 | 4310 | |
| Model 2 | 1 | 0.89 (0.55-1.44) | 1.19 (0.87-1.63) | 0.22 |
| Model 3 | 1 | 0.92 (0.57-1.48) | 1.23 (0.90-1.70) | 0.16 |
| Model 4 | 1 | 0.91 (0.57-1.47) | 1.21 (0.88-1.66) | 0.21 |
| Total fermented dairy | <50 | ≥50-100 | ≥100 | |
| Median intake, g/d | 26 | 73 | 185 | |
| Patients / events (n) | 570/33 | 631/33 | 2200/120 | |
| Person-years | 1807 | 1978 | 6930 | |
| Model 2 | 1 | 0.90 (0.55-1.47) | 0.96 (0.65-1.44) | 0.96 |
| Model 3 | 1 | 0.91 (0.55-1.50) | 0.99 (0.66-1.48) | 0.84 |
| Model 4 | 1 | 0.91 (0.56-1.50) | 1.00 (0.67-1.49) | 0.83 |
| Dairy desserts | <30 | ≥30-60 | ≥60 | |
| Median intake, g/d | 13 | 44 | 87 | |
| Patients / events (n) | 1003/56 | 1040/55 | 1358/75 | |
| Person-years | 3184 | 3276 | 4254 | |
| Model 2 | 1 | 0.93 (0.63-1.37) | 0.98 (0.68-1.42) | 0.99 |
| Model 3 | 1 | 0.94 (0.64-1.39) | 1.03 (0.71-1.50) | 0.78 |
| Model 4 | 1 | 0.95 (0.64-1.40) | 1.04 (0.72-1.51) | 0.74 |

[1]Hazard ratios and 95% confidence intervals from Cox regression. Dairy intakes were adjusted for total energy intake. [2] Linear trends were assessed by treating the median value of each category of dairy as a continuous variable in the models. Model 2 was adjusted for age (y), sex, total energy intake (kcal/d), smoking (3 categories), physical activity (3 categories), educational level (3 categories), and alcohol intake (3 categories). Model 3 was additionally adjusted for dietary intakes (g/d) of whole grains, refined grains, potatoes, fruit, vegetables, total red and processed meat, sugar-sweetened beverages, coffee, and tea. Model 4 was additionally adjusted for other dairy intakes.

**Supplemental Table S5.** HRs (95% CIs) for type 2 diabetes with full-fat milk, full-fat yogurt, liquid fermented dairy, and (ice)-cream consumption without adjustment for BMI in the Alpha Omega Cohort[1].

| | **Dairy consumption** | | |
|---|---|---|---|
| | **No intake** | **Any intake** | **P-value** |
| Full-fat milk | | | |
| Median intake, g/d | 0 | 53 | |
| Patients / events (n) | 3068/171 | 333/15 | |
| Person-years | 9673 | 1041 | |
| Model 2 | 1 | 0.79 (0.46-1.34) | 0.38 |
| Model 3 | 1 | 0.78 (0.46-1.34) | 0.37 |
| Model 4 | 1 | 0.79 (0.46-1.36) | 0.40 |
| | | | |
| Full-fat yogurt | 0 | 15 | |
| Median intake, g/d | 2398/137 | 1003/49 | |
| Patients / events (n) | 7573 | 3142 | |
| Person-years | 1 | 0.84 (0.60-1.17) | 0.30 |
| Model 2 | 1 | 0.84 (0.60-1.17) | 0.31 |
| Model 3 | 1 | 0.85 (0.61-1.19) | 0.36 |
| Model 4 | | | |
| | | | |
| Liquid fermented dairy | | | |
| Median intake, g/d | 0 | 75 | |
| Patients / events (n) | 2108/113 | 1293/73 | |
| Person-years | 6626 | 4088 | |
| Model 2 | 1 | 1.06 (0.79-1.43) | 0.67 |
| Model 3 | 1 | 1.07 (0.80-1.45) | 0.62 |
| Model 4 | 1 | 1.08 (0.80-1.46) | 0.59 |
| | | | |
| (Ice)-cream | 0 | 13 | |
| Median intake, g/d | 1163/70 | 2238/116 | |
| Patients / events (n) | 3667 | 7047 | |
| Person-years | 1 | 0.84 (0.61-1.14) | |
| Model 2 | 1 | 0.86 (0.63-1.17) | 0.27 |
| Model 3 | 1 | 0.86 (0.63-1.18) | 0.34 |
| Model 4 | | | 0.37 |

[1]Hazard ratios and 95% confidence intervals from Cox regression. Full-fat milk, full-fat yogurt, liquid fermented dairy and (ice)-cream) are modelled as any intake vs no intake. Model 2 was adjusted for age (y), sex, total energy intake (kcal/d), smoking (3 categories), physical activity (3 categories), educational level (3 categories), and alcohol intake (4 categories). Model 3 was additionally adjusted for dietary intakes (g/d) of whole grains, refined grains, potatoes, fruit, vegetables, total red and processed meat, sugar-sweetened beverages, coffee, and tea. Model 4 was additionally adjusted for other dairy intakes.

**Supplemental Table S6:** Longitudinal changes in anthropometric outcomes after 3-y of follow-up in 2313 patients from the Alpha Omega Cohort[1].

| | Baseline measurement | Follow-up measurement | Mean difference (95% CIs) | P-value |
|---|---|---|---|---|
| Body weight, kg | 82.24 | 82.08 | 0.16 (-0.02-0.34) | 0.55 |
| BMI, kg/m$^2$ | 27.72 | 27.75 | -0.04 (-0.10-0.03) | 0.06 |
| Waist circumference, cm | 101.33 | 101.50 | -0.18 (-0.42-0.06) | 0.09 |

[1]Changes in anthropometric outcomes over time were estimated in a subsample of the Alpha Omega Cohort that had complete anthropometric measurements after 40 months of follow-up. Mean differences with 95% CIs and p-values were obtained using the Wilcoxon signed rank test.

**Supplemental Table S7.** Beta coefficients (95% CIs) for 3-y changes in anthropometric outcomes according to categories of types of dairy consumption in 2313 patients from the Alpha Omega Cohort[1].

| | **Body weight (kg)** | | | **BMI ($kg/m^2$)** | | | **Waist circumference (cm)** | | |
|---|---|---|---|---|---|---|---|---|---|
| | **β** | **95% CI** | **P-value** | **β** | **95% CI** | **P-value** | **β** | **95% CI** | **P-value** |
| Total dairy | | | | | | | | | |
| <200 g/d | ref | | | ref | | | ref | | |
| ≥200-<400 g/d | 0.36 | -0.09, 0.80 | 0.11 | 0.11 | -0.05, 0.27 | 0.17 | 0.54 | -0.04, 1.12 | 0.06 |
| ≥400 g/d | -0.16 | -0.67, 0.36 | 0.55 | -0.06 | -0.25, 0.13 | 0.55 | 0.04 | -0.64, 0.72 | 0.90 |
| Total milk | | | | | | | | | |
| <50 g/d | ref | | | ref | | | ref | | |
| ≥50-<150 g/d | -0.18 | -0.63, 0.27 | 0.44 | -0.05 | -0.22, 0.11 | 0.53 | -0.16 | -0.75, 0.43 | 0.59 |
| ≥150 g/d | -0.22 | -0.68, 0.23 | 0.33 | -0.05 | -0.21, 0.12 | 0.59 | -0.43 | -1.03, 0.17 | 0.15 |
| Low-fat milk | | | | | | | | | |
| <50 g/d | ref | | | ref | | | ref | | |
| ≥50-<150 g/d | -0.18 | -0.63, 0.27 | 0.42 | -0.07 | -0.24, 0.09 | 0.38 | -0.35 | -0.93, 0.24 | 0.25 |
| ≥150 g/d | -0.23 | -0.69, 0.23 | 0.33 | -0.06 | -0.23, 0.11 | 0.48 | -0.39 | -0.99, 0.21 | 0.20 |
| Full-fat milk | | | | | | | | | |
| No intake | ref | | | ref | | | ref | | |
| Any intake g/d | 0.14 | -0.53, 0.82 | 0.67 | 0.09 | -0.15, 0.34 | 0.44 | 0.74 | -0.14, 1.62 | 0.10 |
| Hard cheese | | | | | | | | | |
| <15 g/d | ref | | | ref | | | ref | | |
| ≥15-<30 g/d | -0.09 | -0.53, 0.35 | 0.68 | -0.06 | -0.22, 0.11 | 0.50 | -0.07 | -0.64, 0.51 | 0.82 |
| ≥30 g/d | -0.08 | -0.55, 0.40 | 0.74 | -0.09 | -0.26, 0.09 | 0.31 | 0.12 | -0.50, 0.74 | 0.70 |
| Total yogurt | | | | | | | | | |
| <25 g/d | ref | | | ref | | | ref | | |
| ≥25-<50 g/d | -0.08 | -0.61, 0.45 | 0.75 | 0.03 | -0.17, 0.22 | 0.77 | -0.44 | -1.13, 0.25 | 0.21 |
| ≥50 g/d | 0.29 | -0.14, 0.72 | 0.19 | 0.07 | -0.08, 0.23 | 0.36 | 0.36 | -0.20, 0.92 | 0.28 |
| Low-fat yogurt | | | | | | | | | |
| <25 g/d | ref | | | ref | | | ref | | |
| ≥25-<50 g/d | -0.40 | -0.97, 0.16 | 0.16 | -0.05 | -0.26, 1.56 | 0.62 | -0.54 | -1.29, 0.20 | 0.15 |
| ≥50 g/d | 0.25 | -0.15, 0.66 | 0.22 | 0.05 | -0.09, 0.20 | 0.47 | 0.37 | -0.17, 0.90 | 0.18 |
| Full-fat yogurt | | | | | | | | | |
| No intake | ref | | | ref | | | ref | | |
| Any intake g/d | 0.03 | -0.38, 0.45 | 0.87 | -0.01 | -0.16, 0.14 | 0.90 | 0.08 | -0.47, 0.63 | 0.76 |
| Liquid fermented dairy | | | | | | | | | |
| No intake | ref | | | ref | | | ref | | |
| Any intake g/d | 0.09 | -0.28, 0.48 | 0.61 | 0.05 | -0.08, 0.19 | 0.45 | 0.24 | -0.25, 0.74 | 0.33 |

| | | | | | | | | | |
|---|---|---|---|---|---|---|---|---|---|
| Total-fermented dairy | | | | | | | | | |
| <50 g/d | ref | | | ref | | | ref | | |
| ≥50-<100 g/d | 0.19 | -0.44, 0.82 | 0.55 | 0.14 | -0.08, 0.38 | 0.21 | 0.35 | -0.48, 1.18 | 0.40 |
| ≥100 g/d | 0.20 | -0.30, 0.72 | 0.42 | 0.05 | -0.13, 0.24 | 0.54 | 0.25 | -0.42, 0.92 | 0.46 |
| (Ice)-cream | | | | | | | | | |
| No intake | ref | | | ref | | | ref | | |
| Any intake g/d | 0.10 | -0.29, 0.51 | 0.59 | 0.10 | -0.04, 0.25 | 0.17 | 0.07 | -0.45, 0.60 | 0.77 |
| Dairy desserts | | | | | | | | | |
| <30 g/d | ref | | | ref | | | ref | | |
| ≥30-<60 g/d | 0.23 | -0.27, 0.72 | 0.36 | 0.07 | -0.11, 0.25 | 0.43 | 0.42 | -0.22, 1.06 | 0.20 |
| ≥60 g/d | 0.33 | -0.15, 0.81 | 0.17 | 0.11 | -0.07, 0.29 | 0.22 | 0.36 | -0.27, 0.99 | 0.20 |

[1]Beta coefficients (β) with 95% confidence intervals and p-values were obtained from multivariable general linear models. Dairy intakes were adjusted for total energy intake except for full-fat milk, full-fat yogurt, liquid fermented dairy and (ice)-cream because of relatively low intake. Multivariable models were adjusted for baseline body weight (kg), BMI (kg/m$^2$) or waist circumference (cm), age (y), sex, total energy intake (kcal/d), smoking (3 categories), physical activity (3 categories), educational level (3 categories), alcohol intake (4 categories), and dietary intakes (g/d) of whole grains, refined grains, potatoes, fruit, vegetables, legumes, total red and processed meat, fish, sugar-sweetened beverages, coffee, tea and other types of dairy.

**Supplemental Table S8:** HRs (95% CIs) for type 2 diabetes according to categories of types of dairy consumption among patients with obesity (n = 685)[1].

| | Categories of dairy consumption (g/d) | | | |
|---|---|---|---|---|
| | Low | Intermediate | High | *P* for trend[2] |
| Total dairy | <200 | ≥200 - 400 | ≥400 | |
| Median intake, g/d | 147 | 287 | 514 | |
| Patients / events (n) | 171/10 | 324/30 | 190/20 | |
| Person-years | 539 | 1007 | 583 | |
| Model 4 | 1 | 1.71 (0.81-3.59) | 2.12 (0.96-4.70) | 0.079 |
| Total milk | <50 | ≥50 - 150 | ≥150 | |
| Median intake, g/d | 18 | 100 | 218 | |
| Patients / events (n) | 247/19 | 222/23 | 216/18 | |
| Person-years | 776 | 678 | 675 | |
| Model 4 | 1 | 1.49 (0.80-2.80) | 1.14 (0.58-2.24) | 0.77 |
| Low-fat milk | <50 | ≥50 - 150 | ≥150 | |
| Median intake, g/d | 16 | 100 | 229 | |
| Patients / events (n) | 288/23 | 197/21 | 200/16 | |
| Person-years | 905 | 598 | 626 | |
| Model 4 | 1 | 1.48 (0.80-2.75) | 1.07 (0.55-2.06) | 0.38 |
| Hard cheese | <15 | ≥15-30 | ≥30 | |
| Median intake, g/d | 10 | 22 | 48 | |
| Patients / events (n) | 265/ 22 | 244/16 | 176/22 | |
| Person-years | 818 | 772 | 539 | |
| Model 4 | 1 | 0.73 (0.36-1.45) | 1.36 (0.73-2.54) | 0.21 |
| Total yogurt | <25 | ≥25-50 | ≥50 | |
| Median intake, g/d | 9 | 38 | 88 | |
| Patients / events (n) | 252/19 | 125/8 | 308/33 | |
| Person-years | 791 | 392 | 946 | |
| Model 4 | 1 | 0.97 (0.41-2.30) | 1.72 (0.93-3.16) | 0.056 |
| Low-fat yogurt | <25 | ≥25-50 | ≥50 | |
| Median intake, g/d | 6 | 38 | 91 | |
| Patients / events (n) | 302/22 | 95/6 | 288/32 | |
| Person-years | 948 | 303 | 878 | |
| Model 4 | 1 | 0.90 (0.35-2.28) | 1.77 (0.99-3.17) | 0.041 |
| Total fermented dairy | <50 | ≥50-100 | ≥100 | |
| Median intake, g/d | 29 | 78 | 192 | |
| Patients / events (n) | 93/7 | 122/8 | 470/45 | |
| Person-years | 295 | 375 | 1459 | |
| Model 4 | 1 | 0.94 (0.32-2.69) | 1.53 (0.66-3.54) | 0.16 |
| Dairy desserts | <30 | ≥30-60 | ≥60 | |

| | | | | |
|---|---|---|---|---|
| Median intake, g/d | 14 | 45 | 87 | |
| Patients /events (n) | 191/10 | 224/23 | 270/27 | |
| Person-years | 611 | 688 | 830 | |
| Model 4 | 1 | 2.53 (1.14-5.62) | 2.58 (1.17-5.69) | 0.041 |

[1]Hazard ratios and 95% confidence intervals from Cox regression. Dairy intakes were adjusted for total energy intake. [2]Linear trends were assessed by treating the median value of each category of dairy as a continuous variable in the models. Model 4 was adjusted for age (y), sex, total energy intake (kcal/d), smoking (3 categories), physical activity (3 categories), educational level (3 categories), alcohol intake (4 categories), and dietary intakes (g/d) of whole grains, refined grains, potatoes, fruit, vegetables, total red and processed meat, sugar-sweetened beverages, coffee, tea, and other dairy.

**Supplemental Table S9:** HRs (95% CIs) for type 2 diabetes according to categories of types of dairy consumption among patients without obesity (n = 2716)[1].

| | Categories of dairy consumption (g/d) | | | |
|---|---|---|---|---|
| | **Low** | **Intermediate** | **High** | ***P* for trend**[2] |
| Total dairy | <200 | ≥200 - 400 | ≥400 | |
| Median intake, g/d | 136 | 283 | 532 | |
| Patients / events (n) | 833/38 | 1235/56 | 648/32 | |
| Person-years | 2620 | 3917 | 2048 | |
| Model 4 | 1 | 1.04 (0.67-1.59) | 1.11 (0.68-1.80) | 0.67 |
| Total milk | <50 | ≥50 - 150 | ≥150 | |
| Median intake, g/d | 10 | 102 | 255 | |
| Patients / events (n) | 1056/46 | 853/42 | 807/38 | |
| Person-years | 3332 | 2688 | 2566 | |
| Model 4 | 1 | 1.12 (0.73-1.71) | 1.07 (0.69-1.66) | 0.92 |
| Low-fat milk | <50 | ≥50 - 150 | ≥150 | |
| Median intake, g/d | 9 | 104 | 258 | |
| Patients / events (n) | 1204/55 | 772/34 | 740/37 | |
| Person-years | 3788 | 2447 | 2350 | |
| Model 4 | 1 | 0.94 (0.61-1.45) | 1.08 (0.71-1.65) | 0.94 |
| Hard cheese | <15 | ≥15-30 | ≥30 | |
| Median intake, g/d | 9 | 21 | 47 | |
| Patients / events (n) | 1132/51 | 930/55 | 654/20 | |
| Person-years | 3578 | 2922 | 2085 | |
| Model 4 | 1 | 1.36 (0.91-2.03) | 0.66 (0.39-1.12) | 0.11 |
| Total yogurt | <25 | ≥25-50 | ≥50 | |
| Median intake, g/d | 8 | 36 | 86 | |
| Patients / events (n) | 1019/51 | 510/23 | 1187/52 | |
| Person-years | 3218 | 1607 | 3761 | |
| Model 4 | 1 | 0.91 (0.55-1.50) | 0.89 (0.60-1.34) | 0.62 |
| Low-fat yogurt | <25 | ≥25-50 | ≥50 | |
| Median intake, g/d | 4 | 37 | 90 | |
| Patients / events (n) | 1253/59 | 378/16 | 1085/51 | |
| Person-years | 3947 | 1206 | 3432 | |
| Model 4 | 1 | 0.87 (0.50-1.53) | 1.01 (0.69-1.50) | 0.89 |
| Total fermented dairy | <50 | ≥50-100 | ≥100 | |
| Median intake, g/d | 25 | 71 | 183 | |
| Patients / events (n) | 477/26 | 509/25 | 1730/75 | |
| Person-years | 1512 | 1603 | 5470 | |
| Model 4 | 1 | 0.89 (0.50-1.56) | 0.80 (0.50-1.28) | 0.36 |
| Dairy desserts | <30 | ≥30-60 | ≥60 | |

| | | | | |
|---|---|---|---|---|
| Median intake, g/d | 13 | 44 | 87 | |
| Patients / events (n) | 812/46 | 816/32 | 1088/48 | |
| Person-years | 2573 | 2588 | 3425 | |
| Model 4 | 1 | 0.65 (0.41-1.05) | 0.81 (0.52-1.26) | 0.50 |

[1]Hazard ratios and 95% confidence intervals from Cox regression. Dairy intakes were adjusted for total energy intake. [2]Linear trends were assessed by treating the median value of each category of dairy as a continuous variable in the models. Model 4 was adjusted for age (y), sex, total energy intake (kcal/d), smoking (3 categories), physical activity (3 categories), educational level (3 categories), alcohol intake (4 categories), and dietary intakes (g/d) of whole grains, refined grains, potatoes, fruit, vegetables, total red and processed meat, sugar-sweetened beverages, coffee, tea, and other dairy.

**Supplemental Table S10:** HRs (95% CIs) for type 2 diabetes according to categories of types of dairy consumption among women (n=680)[1].

| | Categories of dairy consumption (g/d) | | | |
|---|---|---|---|---|
| | **Low** | **Intermediate** | **High** | ***P* for trend[2]** |
| Total dairy | <200 | ≥200 - 400 | ≥400 | |
| Median intake | 163 | 292 | 544 | |
| Patients / events (n) | 124/4 | 342/13 | 214/18 | |
| Person-years | 390 | 1091 | 669 | |
| Model 4 | 1 | 1.11 (0.35-3.54) | 2.64 (0.85-8.19) | 0.016 |
| Total milk | <50 | ≥50 - 150 | ≥150 | |
| Median intake, g/d | 24 | 89 | 212 | |
| Patients / events (n) | 219/10 | 231/6 | 230/19 | |
| Person-years | 698 | 730 | 722 | |
| Model 4 | 1 | 0.72 (0.25-2.05) | 2.26 (0.99-5.15) | 0.023 |
| Low-fat milk | <50 | ≥50 - 150 | ≥150 | |
| Median intake, g/d | 22 | 93 | 229 | |
| Patients / events (n) | 266/13 | 201/4 | 213/18 | |
| Person-years | 845 | 638 | 667 | |
| Model 4 | 1 | 0.43 (0.13-1.37) | 2.00 (0.93-4.28) | 0.61 |
| Hard cheese | <15 | ≥15-30 | ≥30 | |
| Median intake, g/d | 10 | 21 | 46 | |
| Patients / events (n) | 222/13 | 289/14 | 169/8 | |
| Person-years | 690 | 924 | 536 | |
| Model 4 | 1 | 0.89 (0.38-2.10) | 0.79 (0.31-2.05) | 0.65 |
| Total yogurt | <25 | ≥25-50 | ≥50 | |
| Median intake, g/d | 12 | 37 | 91 | |
| Patients / events (n) | 200/11 | 126/5 | 354/19 | |
| Person-years | 624 | 403 | 1123 | |
| Model 4 | 1 | 0.76 (0.24-2.39) | 0.90 (0.40-2.01) | 0.90 |
| Low-fat yogurt | <25 | ≥25-50 | ≥50 | |
| Median intake, g/d | 9 | 38 | 93 | |
| Patients / events (n) | 265/12 | 96/4 | 319/19 | |
| Person-years | 831 | 311 | 1008 | |
| Model 4 | 1 | 0.69 (0.21-2.29) | 1.10 (0.50-2.41) | 0.67 |
| Total fermented dairy | <50 | ≥50-100 | ≥100 | |
| Median intake, g/d | 35 | 73 | 197 | |
| Patients / events (n) | 60/6 | 117/4 | 503/25 | |
| Person-years | 186 | 369 | 1595 | |
| Model 4 | 1 | 0.37 (0.09-1.45) | 0.45 (0.17-1.22) | 0.34 |
| Dairy desserts | <30 | ≥30-60 | ≥60 | |

| | | | | |
|---|---|---|---|---|
| Median intake, g/d | 19 | 46 | 84 | |
| Patients / events (n) | 101/8 | 215/12 | 364/15 | |
| Person-years | 316 | 674 | 1159 | |
| Model 4 | 1 | 0.58 (0.21-1.58) | 0.48 (0.18-1.25) | 0.18 |

[1]Hazard ratios and 95% confidence intervals from Cox regression. Dairy intakes were adjusted for total energy intake. [2]Linear trends were assessed by treating the median value of each category of dairy as a continuous variable in the models. Model 4 was adjusted for age (y), total energy intake (kcal/d), smoking (3 categories), physical activity (3 categories), educational level (3 categories), alcohol intake (4 categories), BMI (kg/m$^2$), and dietary intakes (g/d) of whole grains, refined grains, potatoes, fruit, vegetables, total red and processed meat, sugar-sweetened beverages, coffee, tea, and other dairy.

**Supplemental Table S11:** HRs (95% CIs) for type 2 diabetes according to categories of types of dairy consumption among men (n=2721)[1].

| | Categories of dairy consumption (g/d) | | | |
|---|---|---|---|---|
| | Low | Intermediate | High | *P* for trend[2] |
| Total dairy | <200 | ≥200 - 400 | ≥400 | |
| Median intake, g/d | 136 | 281 | 522 | |
| Patients / events (n) | 880/44 | 1217/73 | 624/34 | |
| Person-years | 2770 | 3832 | 1963 | |
| Model 4 | 1 | 1.22 (0.83-1.80) | 1.09 (0.69-1.73) | 0.75 |
| Total milk | <50 | ≥50 - 150 | ≥150 | |
| Median intake, g/d | 8 | 104 | 255 | |
| Patients / events (n) | 1084/55 | 844/59 | 793/37 | |
| Person-years | 3410 | 2636 | 2519 | |
| Model 4 | 1 | 1.37 (0.95-1.99) | 0.91 (0.60-1.40) | 0.39 |
| Low fat milk | <50 | ≥50 - 150 | ≥150 | |
| Median intake, g/d | 7 | 106 | 255 | |
| Patients / events (n) | 1226/65 | 768/51 | 727/35 | |
| Person-years | 3849 | 2408 | 2308 | |
| Model 4 | 1 | 1.24 (0.85-1.80) | 0.90 (0.59-1.37) | 0.63 |
| Hard cheese | <15 | ≥15-30 | ≥30 | |
| Median intake, g/d | 9 | 21 | 48 | |
| Patients / events (n) | 1175/60 | 885/57 | 661/34 | |
| Person-years | 3707 | 2770 | 2088 | |
| Model 4 | 1 | 1.24 (0.85-1.80) | 0.94 (0.61-1.45) | 0.72 |
| Total yogurt | <25 | ≥25-50 | ≥50 | |
| Median intake, g/d | 7 | 36 | 85 | |
| Patients / events (n) | 1071/59 | 509/26 | 1141/66 | |
| Person-years | 3384 | 1596 | 3584 | |
| Model 4 | 1 | 0.96 (0.60-1.54) | 1.11 (0.77-1.60) | 0.52 |
| Low-fat yogurt | <25 | ≥25-50 | ≥50 | |
| Median intake, g/d | 4 | 37 | 89 | |
| Patients / events (n) | 1290/69 | 377/18 | 1054/64 | |
| Person-years | 4064 | 1199 | 3302 | |
| Model 4 | 1 | 0.92 (0.54-1.55) | 1.20 (0.84-1.71) | 0.28 |
| Total fermented dairy | <50 | ≥50-100 | ≥100 | |
| Median intake, g/d | 25 | 73 | 180 | |
| Patients / events (n) | 510/27 | 514/29 | 1697/95 | |
| Person-years | 1621 | 1609 | 5335 | |
| Model 4 | 1 | 1.02 (0.59-1.74) | 1.07 (0.69-1.67) | 0.71 |
| Dairy desserts | <30 | ≥30-60 | ≥60 | |

| | | | | |
|---|---|---|---|---|
| Median intake, g/d | 12 | 44 | 88 | |
| Patients / events (n) | 902/48 | 825/43 | 994/60 | |
| Person-years | 2867 | 2602 | 3096 | |
| Model 4 | 1 | 0.96 (0.63-1.48) | 1.20 (0.80-1.80) | 0.32 |

[1]Hazard ratios and 95% confidence intervals from Cox regression. Dairy intakes were adjusted for total energy intake. [2]Linear trends were assessed by treating the median value of each category of dairy as a continuous variable in the models. Model 4 was adjusted for age (y), total energy intake (kcal/d), smoking (3 categories), physical activity (3 categories), educational level (3 categories), alcohol intake (4 categories), BMI (kg/m$^2$), and dietary intakes (g/d) of whole grains, refined grains, potatoes, fruit, vegetables, total red and processed meat, sugar-sweetened beverages, coffee, tea, and other dairy.
